# Immune responses to SARS-CoV-2 in vaccinated patients receiving checkpoint blockade immunotherapy for cancer

**DOI:** 10.3389/fimmu.2022.1022732

**Published:** 2022-12-13

**Authors:** Alexander Piening, Emily Ebert, Niloufar Khojandi, Elise Alspach, Ryan M. Teague

**Affiliations:** Department of Molecular Microbiology and Immunology, School of Medicine, Saint Louis University, St. Louis MO, United States

**Keywords:** immune checkpoint blockade (ICB), cancer, COVID - 19, vaccination, immunotherapy

## Abstract

Vaccination against SARS-CoV-2 has been successful in protecting patients with cancer from severe infections, but how immune responses against COVID-19 vaccination interact with those elicited during cancer immunotherapy has not been fully described. Immune checkpoint blockade (ICB) disrupts inhibitory pathways in immune cells to improve function and induce tumor immunity but can often cause serious immune related adverse events (IRAEs). Because COVID-19 vaccination and ICB both boost immune responses, it is imperative to understand if combining these regimens causes synergistic enhancement of the immune system. Specifically, whether ICB impacts anti-vaccine immunity in previously vaccinated patients is important since a large percentage of newly diagnosed cancer patients eligible for immunotherapy will have already been vaccinated against COVID-19. To address this, we investigated the influence of ICB on SARS-CoV-2-spike protein (SP) antibody titers and T cell responses in cancer patients previously vaccinated against COVID-19. Human blood samples were collected from 29 vaccinated patients and 12 unvaccinated control patients at baseline (prior to ICB) and following two rounds of ICB infusion. Anti-SARS-CoV-2-SP IgG titers and T cell responses were quantified. Compared to responses at baseline, there was no significant difference in these immune responses after immunotherapy in vaccinated individuals (P=0.4583, P=0.4571, respectively). We interpret these results as evidence that ICB immunotherapy does not significantly enhance SARS-CoV-2-specific antibody titers or T cell responses. Although our study lacks corresponding IRAE rates, the results provide humoral and cellular immunological data that support recent reports documenting the clinical safety and efficacy of COVID-19 vaccination in patients receiving ICB. Additional longitudinal prospective studies, such as the VOICE study (ClinicalTrials.gov identifier NCT04715438) and CAPTURE study (ClinicalTrials.gov identifier NCT03226886), are warranted and will provide broader safety and immunological data defining the effect of systemic cancer therapies on COVID-19 immunity.

## Introduction

The coronavirus disease 2019 (COVID-19) pandemic has had a profound negative impact on human health worldwide. According to current CDC data, approximately 28% of the United States population has been infected by severe acute respiratory syndrome coronavirus 2 (SARS-CoV-2), resulting in over 1 million deaths (1). Fortunately, the development of efficacious vaccines has been successful in preventing severe infection in the majority of vaccinated individuals (2, 3). Vaccination against COVID-19 is particularly important for patients with cancer due to generally impaired immunity in this cohort, leaving them vulnerable to severe disease from infection (4–7). While vaccination is crucial to protecting cancer patients against COVID-19 infection, potential interactions between the immune responses generated by vaccines and those elicited by immune-modulatory cancer therapies have yet to be fully elucidated.

Immune checkpoint blockade (ICB) treatment for cancer works by blocking inhibitory molecules on immune cells to improve their function and induce tumor immunity. Because the goal of vaccination is also to boost immune responses, albeit against viral antigens and not tumor cells, there is a concerted research effort to better understand the safety and immunological implications of ICB treatment on both previously vaccinated cancer patients and patients receiving initial COVID-19 vaccination while being treated with ICB (8–10).

Although ICB immunotherapy is often effective at suppressing tumor progression, ICB is also associated with a range of immune related adverse events (IRAEs) due to potent systemic reinvigoration of the immune system (11–13). For this reason, it is critical to understand whether concurrent COVID-19 vaccination and ICB increase risk of IRAEs due to synergistic enhancement of responding immune cells. Conversely, there is the distinct possibility that ICB could boost the efficacy of COVID-19 vaccination, as recently suggested with seasonal influenza vaccination (14, 15). Emerging evidence has demonstrated the relative efficacy and safety of COVID-19 vaccination in patients already receiving systemic cancer therapy, including ICB (16–19). However, the immunological impact of ICB treatment in cancer patients already vaccinated against COVID-19 has not been investigated. This is of particular importance as a large percentage of newly diagnosed cancer patients eligible for immunotherapy will have already been vaccinated against COVID-19. To address this outstanding clinical question, we obtained serum and peripheral blood mononuclear cells (PBMCs) before and after anti-PD-1/L1 ICB immunotherapy from a cohort of patients with diverse cancers that were previously vaccinated against COVID-19. The effects of ICB treatment on SARS-CoV-2 specific antibody titers and T cell responses were then assessed.

## Materials and methods

### Study participants

**Figure 1:**
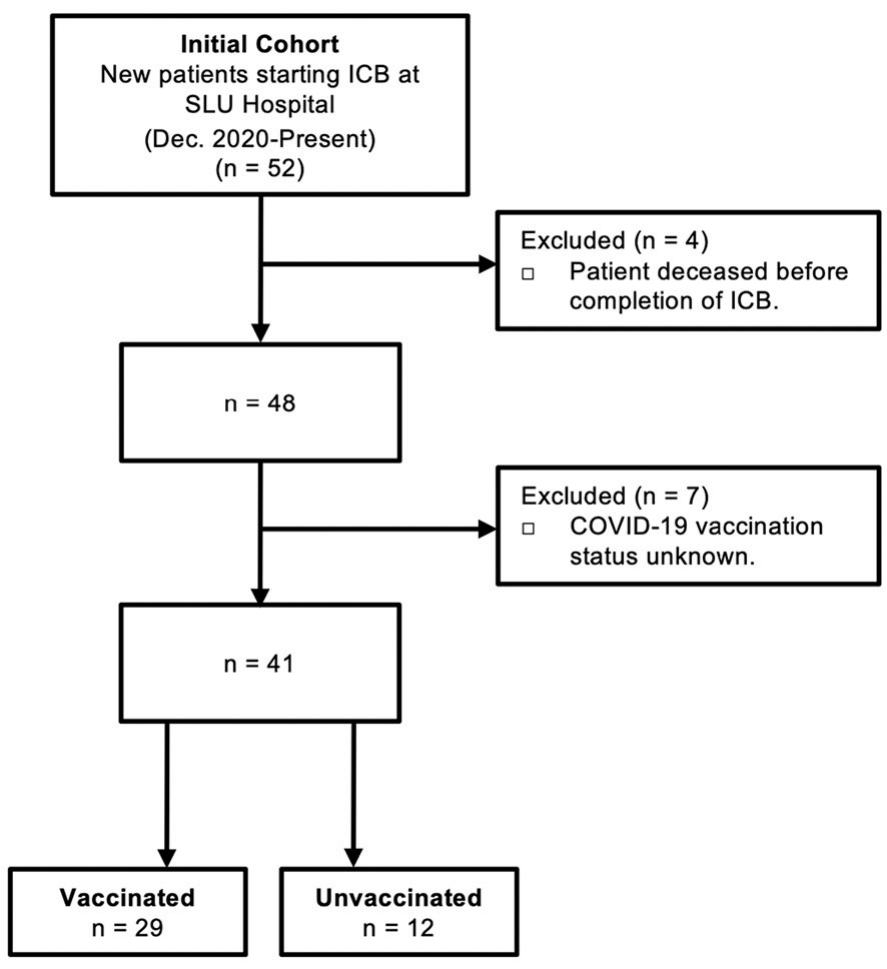
Criteria for study inclusion. Consolidated Standard of Reporting Trials (CONSORT) diagram describing the selection of COVID-19 vaccinated patients and control unvaccinated patients that received immune checkpoint blockade (ICB) immunotherapy for cancer at Saint Louis University (SLU) Hospital from December 2020 to present.

Human blood was obtained from 52 patients with diverse cancer types receiving anti-PD-1/L1 ICB therapy at Saint Louis University Hospital (St. Louis, MO). Patients did not receive anti-CTLA-4 either alone or in combination with other ICB. Patient data regarding concomitant receipt of conventional chemotherapy was not available for this cohort, nor was data regarding administration of corticosteroids for treatment of possible immune-related adverse events. Blood was collected at baseline (prior to ICB) and following administration of two rounds of ICB infusion (post-ICB). The post-ICB blood sample was collected 2-3 weeks after the 2^nd^ ICB infusion (4-6 weeks after first treatment). All participants provided written informed consent in accordance with the Declaration of Helsinki. Screening criteria for study inclusion are outlined in **Figure 1**.

### Blood processing

Whole blood was collected in a Beckton Dickenson Vacutainer with 158 USP units sodium heparin. Blood was centrifuged at 1500 G for 10-minutes to separate cells from plasma. Plasma was collected and stored at -80 degrees Celsius. The cellular fraction was resuspended and overlaid onto Ficoll-Paque PLUS (GE Healthcare Life Sciences). Peripheral blood mononuclear cells (PBMCs) were separated from red blood cells by centrifugation at 2000 RPM for 30-minutes. PBMCs were removed, washed 2-times in phosphate buffered saline (PBS), resuspended in freezing media consisting of 10% DMSO, 40% fetal bovine serum, 50% RPMI 1640 media (Gibco), frozen gradually at -80°C, and stored in liquid nitrogen prior to analysis.

### Quantification of human SARS-CoV-2 spike IgG antibody titers

SARS-CoV-2 spike protein IgG antibody titers were quantified in duplicate from patient plasma using the Invitrogen Human SARS-CoV-2-Spike (trimer) IgG ELISA Kit (Cat#: BMS2325) according to the manufacturer’s protocol.

### Quantification of human T cell responses to SARS-CoV-2 spike protein

Human T cell responses to SARS-CoV-2 spike protein were quantified using the Immunospot Human IFNγ Single-Color Enzymatic ELISPOT Assay with precoated 96-well strip plates according to the manufacturer’s protocol. PBMCs were plated in precoated Immunospot 96-well strip plate in triplicate at a concentration of 4x10^5^ cells/well and incubated with control peptide or a peptide pool spanning the SARS-CoV-2 spike protein (4μg/mL) obtained from BEI Resources (Cat#: NR-52402). PBMCs stimulated with phytohemagglutinin-P (PHA) served as a positive control for the assay. After 24-hours of incubation, IFNγ spots were quantified using the Immunospot CTL S6 Universal-V analyzer.

### Statistical analysis

Patient characteristics were compared using chi-squared tests for categorical variables with standard deviation (SD) (Prism 9.0, GraphPad Software). Differences in antibody titers and T cell responses were determined using an unpaired, two-tailed nonparametric Mann-Whitney *U* test with standard error of the mean (SEM) using Prism 9.0. Linear regression analysis to assess correlation between two parameters was performed by calculating the Pearson value (r) and the corresponding *P* value using Prism 9.0. Unpaired, one-way Kruskal-Wallis analyses of variance were used to compare responder, non-responder, and negative responder subgroups (Prism 9.0).

## Results

The goal of this study was to define the impact of anti-PD-1/L1 immunotherapy on antibody titers and T cell responses against the SARS-CoV-2 spike protein in patients that received COVID-19 vaccination prior to immunotherapy. Peripheral blood was collected at baseline (prior to ICB) and after two rounds of ICB infusions (post-ICB), approximately 4-6 weeks after initiation of ICB treatment. Of an initial 52 patients, a total of 29 vaccinated participants were identified and included in this study. Unvaccinated patients (n=12) that received ICB treatment for cancer during this same time period served as controls. Patients that deceased before completion of ICB and those with unknown vaccination status were excluded from the study. Criteria for study inclusion are outlined in **Figure 1**.

A description of the 29 vaccinated patient characteristics can be found in **Table 1**. Of the 29 patients, there was a higher frequency of males (n=20) compared to females (n=9) (*P*=0.0411). The average age of patients was 67.08 (+/- 7.48) years, and the average body mass index (BMI) was 29.29 (+/- 5.57). There was a higher frequency of white patients (n=19) compared to black (n=8) and Asian (n=2) (*P*=0.0009). Patients were receiving ICB therapy for diverse cancer types including small cell lung cancer, non-small cell lung cancer, squamous cell lung cancer, lung adenocarcinoma, hepatocellular carcinoma, esophageal cancer, among others. The most common ICB treatment was pembrolizumab (n=15), compared to durvalumab (n=5), nivolumab (n=4), and atezolizumab (n=5) (*P*=0.0110). No patients in the study received anti-CTLA-4 therapy either alone or in combination with other ICB. Of the 29 patients, 11 had received 2 doses of the Moderna (mRNA-1273) vaccine and 18 had received 2 doses of the Pfizer BioNTech (BNT162b2) vaccine (*P*=0.1936). The average time from vaccination to ICB treatment was 153.76 days (+/-109.44).

**Table 1:** Description of vaccinated patient characteristics.

	N=29	P-value
Sex		0.0411
Male	20	
Female	9	
Age (years)	67.08 (+/-7.48)	
Race		0.0009
White	19	
Black	8	
Asian	2	
BMI	29.29 (+/-5.57)	
Type of Vaccine		0.1936
Moderna	11	
Pfizer	18	
Type of Cancer		0.7704
Small Cell Lung Cancer	4	
Non-small Cell Lung Cancer	6	
Squamous Cell Lung Cancer	4	
Lung Adenocarcinoma	3	
Hepatocellular Carcinoma	3	
Esophageal	2	
Other^*^	7	
Type of Immunotherapy		0.0110
Pembrolizumab	15	
Durvalumab	5	
Nivolumab	4	
Atezolizumab	5	
Time Since Vaccination (Days)	153.76 (+/-109.44)	

^*^Other includes pancreatic cancer, brain cancer, renal cell carcinoma, sarcoma, metastatic papillary thyroid cancer, head/neck cancer.

**Figure 2:**
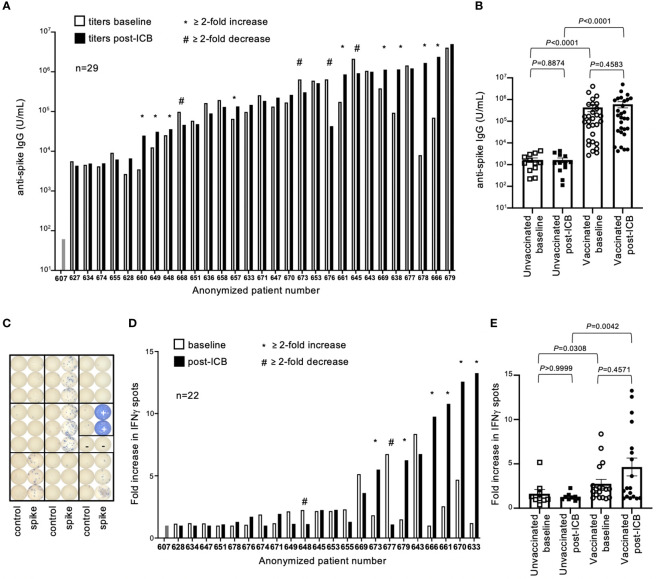
SARS-CoV-2 spike protein antibody titers and T cell responses in vaccinated patients before and after immune checkpoint blockade. **(A)** Antibody (IgG) titers to SARS-CoV-2 spike protein were quantified for 29 previously vaccinated patients at baseline (open bars) and following two rounds of ICB infusion (closed bars). Antibody titers were compared to a pre-COVID control (patient #607). Each bar represents data from an individual patient, with responders (*) and negative responders (#) indicated. **(B)** Vaccinated patient titers were evaluated as a group at baseline and post-ICB and compared to unvaccinated patients that received ICB in the same timeframe. Bars represent average titer, and error bars are SEM for each cohort. Each point represents data from an individual patient. **(C)** Image of ELISPOT plate provides visual demonstration of differences in IFNγ spots generated from stimulation of PBMCs with either control peptide, specific peptide spanning the SARS-CoV-2 spike protein, or PHA positive control (+). **(D)** Quantification of T cell responses to SARS-CoV-2 spike protein from 22 individual vaccinated patients at baseline (open bars) and post-ICB (closed bars), **(E)** grouped by treatment cohort, and compared to unvaccinated patients. Bars represent average fold increase, and error bars are SEM for each cohort. Each point represents data from an individual patient. Exact *P* values are provided for the bracketed groups.

Antibody (IgG) titers to SARS-CoV-2 spike protein (SP), the immunogen encoded in both vaccine formulas, were quantified by ELISA. Antibody titers varied by several orders of magnitude throughout the vaccinated patient cohort, both at baseline and post-ICB (**Figure 2A**). While subsets of patients experienced increased (n=9) or decreased (n=4) titers after ICB, defined as a ≥2-fold change, the majority (n=16) showed no difference in SP-antibody levels after immunotherapy (**Figure 2B**). To investigate potential differences between those patients whose antibody titers increased (≥2-fold increase, “responders”), decreased (≥2-fold decrease, “negative responders”), or remained unchanged (<2-fold change, “non-responders”) following ICB, a subgroup analysis comparing the patient-specific characteristics of these groups was performed. There were no statistical differences in the sex, age, BMI, type of vaccine, type of cancer, type of immunotherapy, or time since vaccination between the responder, non-responder, and negative responder groups (**Supplementary Table 1**). These data suggest that changes, or lack thereof, in antibody titers after ICB are likely the result of patient-specific factors not generalizable to all participants given the information provided in this study. In contrast, antibody titers between vaccinated and unvaccinated patients were intuitively different, with vaccinated patients having significantly higher antibody titers at both baseline and post-ICB compared to the control unvaccinated cohort (*P*<0.0001 and *P*<0.0001, respectively) (**Figure 2B**). Of note, there was a range of antibody titers above the limit of detection in the unvaccinated patient cohort, suggesting that while these patients were not vaccinated, they had likely experienced natural SARS-CoV-2 exposure and/or infection during the pandemic, although this information was not available at the time of our study (**Figure 2B**). Considering pooled data from baseline and post-ICB, immunotherapy did not significantly alter antibody titers for either vaccinated (*P*=0.4583) or unvaccinated cohorts (*P*=0.8874), indicating no underlying immunological enhancement of SARS-CoV-2 antibody responses in vaccinated patients receiving ICB immunotherapy for cancer (**Figure 2B**).

To assess T cell responses against SARS-CoV-2 SP in patients, peripheral blood mononuclear cells (PBMCs) were stimulated with an overlapping peptide pool spanning the spike protein, and IFNγ production was measured by ELISPOT assay 24-hours later (**Figure 2C**). Of the 29 patients, 22 had sufficient numbers of preserved PBMCs for testing. Responses were defined as fold changes in the number of IFNγ spots elicited by specific SARS-CoV-2 peptides compared to control peptides. As defined by a change greater than 2-fold relative to baseline, subsets of patients showed increased T cell responses (n=6) and decreased T cell responses (n=2) after ICB, whereas the majority of patients (n=15) showed no changes in response to ICB (**Figure 2D**). To investigate potential differences between responder, negative responder, and non-responder groups, as defined previously, a subgroup analysis comparing the patient-specific characteristics of these groups was performed. There were no statistical differences in the sex, age, BMI, type of vaccine, type of cancer, type of immunotherapy, or time since vaccination between the responder, non-responder, and negative responder groups for T cell responses (**Supplementary Table 2**). Similar to antibody titers, these results suggest that rare changes in SP-specific T cell responses after ICB are likely unique to each patient and not generalizable to all participants based on the information available in this study. As observed with antibody responses, vaccinated patients had significantly higher T cell responses to spike protein at baseline and post-ICB compared to unvaccinated patients (*P*=0.0308 and *P*=0.0042, respectively) (**Figure 2E**). When assessed as a group, ICB treatment did not significantly impact SP-specific T cell responses based on changes from baseline to post-ICB for either the vaccinated (*P*=0.4571) or the unvaccinated (*P*>0.9999) cohorts, indicating no underlying immunological enhancement of T cell responses against SARS-CoV-2 SP in vaccinated patients during immunotherapy (**Figure 2E**).

**Figure 3:**
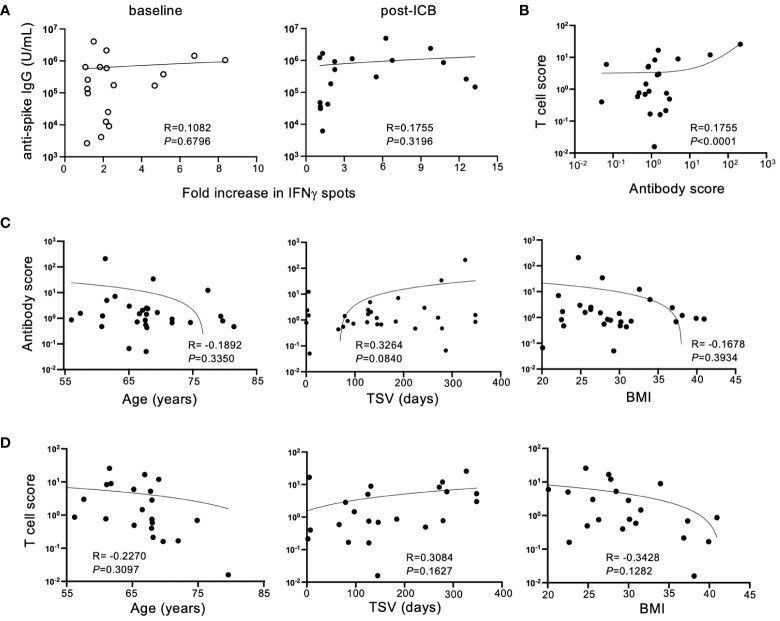
Humoral and cellular immune responses to SARS-CoV-2 SP after ICB correlate in a patient-specific, but not generalizable, manner. **(A)** Correlation between SARS-CoV-2 spike protein antibody titers and T cell responses at baseline (prior to ICB) and following two rounds of ICB infusion. **(B)** Antibody and T cell scores were generated for the vaccinated patient cohort by dividing the post-ICB value (titer or T cell activity) by the baseline value, respectively. Correlations between antibody score and T cell score in the vaccinated patient cohort were determined. **(C)** Correlation between antibody score of vaccinated patients (n=29) and age, time since vaccination (TSV), and BMI. **(D)** Correlation between T cell score of vaccinated patients (n=22) and age, time since vaccination, and BMI. Each point represents data from an individual patient and inset numbers indicate the *r* values for each dataset along with corresponding *P* values.

To determine if antibody titers and T cell responses against SARS-CoV-2 SP corresponded for each patient within the vaccinated cohort, antibody titers were compared to IFNγ responses for individual patients at baseline and post-ICB. Despite the expectation that adaptive immune responses would be concerted, there was no association between individual patient IgG titers and T cell responses either at baseline or post-ICB (**Figure 3A**), suggesting that for vaccinated patients with high serum antibody titers, antigen-reactive T cells in the blood are not always present in high numbers, and vice versa. Next, to determine whether changes in adaptive immunity induced by ICB showed a correlation, an antibody response score and a T cell response score was generated for each individual patient. Scores were calculated by taking post-ICB titers or T cell activity and dividing that value by the corresponding values at baseline. Thus, for example, if a patient had a two-fold increase in antibody titer from baseline to post-ICB, that patient’s antibody response score would be 2, and similarly for a 2-fold increase in T cell response. Whereas, if a patient had a two-fold decrease in antibody titer from baseline to post-ICB, their response score would be a 0.5, and similarly for a 2-fold decrease in T cell response. Analyzing responses to ICB in this way revealed a significant correlation (*P <*0.0001) between antibody scores and T cell response scores within individual patients (**Figure 3B**). This indicates that T cell and antibody responses tended to change in parallel for patients from baseline to post-ICB. Thus, patients who experienced an increase in anti-SARS-CoV-2 IgG titers after immunotherapy were likely to have increased peripheral T cell activity, and vice versa (**Figure 3B**).

To define the reason for higher adaptive immune responses against SARS-CoV-2 after immunotherapy in some patients but not others, T cell scores and antibody scores, as defined above, were compared to patient-specific characteristics. Immunotherapy-driven antibody scores were unaffected by patient age, time since vaccination (TSV) or BMI (**Figure 3C**). This was also true for ICB-induced T cell scores (**Figure 3D**). Although not statistically significant, these immune responses induced by immunotherapy trended downward with advancing age and higher BMI. In contrast, increased time since vaccination seemed to track with higher B and T cell responses against COVID SP following ICB, but this relationship did not reach the threshold of statistical significance (*P*=0.0840) (**Figure 3C**). This result is counterintuitive considering immune responses to vaccination are expected to wane over time. It is possible that this is due to responses that were generated by booster vaccination or natural exposures which accumulated over time, but this information that was not available in this study.

## Discussion

The concurrent use of COVID-19 vaccines with checkpoint blockade immunotherapy has been a topic of heightened interest due to the hypothesized potential to either elicit increased rates of IRAEs in vaccinated patients with cancer or, potentially, boost immunological responses to the vaccine, offering heightened protection to this vulnerable patient populations (8–10, 16–19). Emerging clinical studies have largely concluded that COVID-19 vaccination is both safe and efficacious in patients already receiving ICB, but the impact of ICB on previously vaccinated cancer patients was unknown at the initiation of our study (16–18). Importantly, SARS-CoV-2 infection has been linked to increased expression of exhaustion markers such as PD-1 on SARS-CoV-2-specific T cells, implying that ICB can induce acute reactivation of these cells. However, whether specific immune responses against COVID-19 vaccination are augmented in vaccinated patients subsequently treated with ICB has not been reported (20–23).

Considering that the theoretical synergism between COVID-19 vaccination and ICB has been the foundation for many recent clinical studies investigating patient safety and vaccine efficacy, our study sought to determine if ICB treatment impacts SARS-CoV-2-specific immune responses in previously vaccinated patients with cancer. Comparing baseline and post-ICB, our results demonstrated that anti-PD-1/L1 immunotherapy had no significant influence on SARS-CoV-2 SP antibody titers or T cell responses in a cohort of 29 vaccinated patients with diverse cancers. Subsets of patients had increased responses while others had decreased responses after ICB, but the majority of patients had similar antibody titers and T cell responses before and after ICB treatment. Subgroup analysis failed to delineate any patient-specific characteristics that could drive differences in these responses, including important metrics such as cancer type, vaccine type, treatment regimen, and time since vaccination. Together, these data support the hypothesis that ICB treatment in COVID-19 vaccinated individuals does not result in immunological enhancement of SARS-CoV-2 specific immune responses.

One limitation of our study is lack of access to COVID-19 exposure history and booster vaccination data for our cohort. If a patient had received a vaccine booster or experienced a COVID-19 exposure prior to or during ICB treatment, this could potentially boost their immune responses and confound interpretation of post-ICB antibody and T cell metrics. While this confounding effect is possible, the large majority of patients and the pooled data showed no difference in anti-vaccine immunity post-ICB, suggesting there is no confounding effect on the study population as a whole. Conversely, response to vaccination could potentially be dampened in patients with impaired performance status, baseline immunosuppression, and in patients receiving cytotoxic chemotherapy or other immunosuppressive drugs (24, 25). Given that certain patients in our cohort could have received concomitant chemotherapy or corticosteroids for treatment of IRAEs, we acknowledge this lack of data as a limitation of the study. However, while this limitation is possible for individual patients, analysis of the vaccinated population as a whole suggests robust vaccine-mediated immunity that is not significantly altered by ICB treatment, mitigating this potentially confounding factor of the study. Nevertheless, inclusion of exposure data, booster administration, performance status, and concurrent treatments are important for future prospective studies. Additionally controlled prospective studies, such as those outlined in the VOICE study (‘vaccination against COVID in cancer’, ClinicalTrials.gov identifier: NCT04715438) and the CAPTURE study (‘COVID-19 Antiviral Response in a Pan-tumor Immune Study’ ClinicalTrials.gov identifier: NCT03226886), will help to further elucidate the impact of these variables on responses to COVID-19 vaccination in cancer patients receiving ICB (8, 9).

An additional limitation of this study is the lack of corresponding data on rates of IRAEs in our patient cohort. While this is a significant limitation, our results provide discrete cellular and humoral immunological evidence that reinforces other recent clinical studies demonstrating the safety and efficacy of combined COVID-19 vaccination with ICB treatment in cancer patients (16–19). Given that our study investigated the impact of ICB on anti-COVID-19 immune responses after two ICB infusions, analysis of anti-COVID-19 immunity at later timepoints following additional treatments could be informative. Furthermore, no patients in this study were receiving anti-PD-1/L1 and anti-CTLA-4 dual therapy. Follow-up investigation in patient cohorts receiving this combination ICB would add additional evidence towards delineating the impact of ICB on responses to COVID-19 vaccination. Because this is a small cohort study, larger longitudinal studies, such as the VOICE and CAPTURE studies, are needed to provide broader evidence regarding the safety and immunological implications of COVID-19 vaccination in cancer patients receiving systemic therapies, particularly immunotherapy.

## Data availability statement

The original contributions presented in the study are included in the article/**Supplementary Material**. Further inquiries can be directed to the corresponding author.

## Ethics statement

The studies involving human participants were reviewed and approved by Saint Louis University Institutional Review Board Approval. The patients/participants provided their written informed consent to participate in this study.

## Author contributions

Acquisition, analysis, or interpretation of data – AP, EE, and NK. Drafting of manuscript – AP, EE, EA, and RT. Statistical analysis – AP. Critical revision of the manuscript – AP, EE, NK, EA, RT. Obtained funding – RT. Conception of design – AP, EA, and RT. All authors contributed to the article and approved the submitted version.
