# Supplementary material for: Immune responses to SARS-CoV-2 in vaccinated patients receiving checkpoint blockade immunotherapy for cancer
Source: Front Immunol. 2022 Dec 13;13:1022732. doi: 10.3389/fimmu.2022.1022732 (PMC9792507; doi:10.3389/fimmu.2022.1022732)
Supplement: Supplementary file 1 [file Table_1.docx]

**Supplementary Table 1.** Comparison of patient-specific characteristics for SARS-CoV-2 spike protein antibody responders (≥2-fold increase), non-responders (<2-fold change), and negative responders (≥2-fold decrease) comparing baseline to post-immune checkpoint blockade.

|  | **Responders** | **Non-responders** | **Negative Responders** |  |
| --- | --- | --- | --- | --- |
|  | **N=9** | **N=16** | **N=4** | **P-value** |
| Sex |  |  |  | 0.3423 |
| Male | 5 | 13 | 2 |  |
| Female | 4 | 3 | 2 |  |
| Age (years) | 66.64 (+/-4.93) | 66.86 (+/-8.95) | 68.76 (+/-8.94) | 0.9640 |
| Race |  |  |  | 0.1448 |
| White | 3 | 13 | 3 |  |
| Black | 6 | 2 | 0 |  |
| Asian | 0 | 1 | 1 |  |
| BMI | 28.39 (+/-4.92) | 30.80 (+/-5.81) | 25.98 (+/-5.36) | 0.3426 |
| Type of Vaccine |  |  |  | 0.2319 |
| Moderna | 4 | 7 | 0 |  |
| Pfizer | 5 | 9 | 4 |  |
| Type of Cancer |  |  |  | 0.3614 |
| Small Cell Lung Cancer | 2 | 1 | 1 |  |
| Non-small Cell Lung Cancer | 3 | 2 | 1 |  |
| Squamous Cell Lung Cancer | 0 | 4 | 0 |  |
| Lung Adenocarcinoma | 2 | 0 | 1 |  |
| Hepatocellular Carcinoma | 0 | 3 | 0 |  |
| Esophageal | 0 | 1 | 1 |  |
| Other^*^ | 2 | 5 | 0 |  |
| Type of Immunotherapy |  |  |  | 0.3538 |
| Pembrolizumab | 3 | 9 | 3 |  |
| Durvalumab | 3 | 1 | 1 |  |
| Nivolumab | 1 | 3 | 0 |  |
| Atezolizumab | 2 | 3 | 0 |  |
| Time Since Vaccination (Days) | 158.67 (+/-113.68) | 145.67 (+/-106.12) | 214 (+/-102.56) | 0.5284 |

^*^Other includes pancreatic cancer, brain cancer, renal cell carcinoma, sarcoma, metastatic papillary thyroid cancer, head/neck cancer.

All P-values were generated using Kruskal-Wallis tests.

**Supplementary Table 2.** Comparison of patient-specific characteristics for SARS-CoV-2 spike protein T cell responders (≥2-fold increase), non-responders (<2-fold change), and negative responders (≥2-fold decrease) comparing baseline to post-immune checkpoint blockade.

|  | **Responders** | **Non-responders** | **Negative Responders** |  |
| --- | --- | --- | --- | --- |
|  | **N=6** | **N=14** | **N=2** | **P-value** |
| Sex |  |  |  | 0.3082 |
| Male | 5 | 7 | 2 |  |
| Female | 1 | 7 | 0 |  |
| Age (years) | 62.68 (+/-4.23) | 66.15 (+/-8.04) | 67.66 (+/-0.14) | 0.3296 |
| Race |  |  |  | 0.8289 |
| White | 4 | 9 | 2 |  |
| Black | 1 | 5 | 0 |  |
| Asian | 1 | 0 | 0 |  |
| BMI | 29.22 (+/-2.92) | 30.43 (+/-7.13) | 29.23 (+/-1.08) | 0.9831 |
| Type of Vaccine |  |  |  | >0.9999 |
| Moderna | 3 | 7 | 1 |  |
| Pfizer | 3 | 7 | 1 |  |
| Type of Cancer |  |  |  | 0.5790 |
| Small Cell Lung Cancer | 0 | 4 | 0 |  |
| Non-small Cell Lung Cancer | 2 | 2 | 0 |  |
| Squamous Cell Lung Cancer | 1 | 2 | 1 |  |
| Lung Adenocarcinoma | 0 | 2 | 0 |  |
| Hepatocellular Carcinoma | 0 | 1 | 1 |  |
| Esophageal | 1 | 0 | 0 |  |
| Other^*^ | 2 | 3 | 0 |  |
| Type of Immunotherapy |  |  |  | 0.7445 |
| Pembrolizumab | 4 | 5 | 1 |  |
| Durvalumab | 0 | 5 | 0 |  |
| Nivolumab | 1 | 2 | 0 |  |
| Atezolizumab | 1 | 2 | 1 |  |
| Time Since Vaccination (Days) | 140.43 (+/-95.02) | 217.83 (+/-128.16) | 213.5 (+/-190.21) | 0.4403 |

^*^Other includes pancreatic cancer, brain cancer, renal cell carcinoma, sarcoma, metastatic papillary thyroid cancer, head/neck cancer.

All P-values were generated using Kruskal-Wallis tests.
